# Developmental plasticity research in evolution and human health: Response to commentaries

**DOI:** 10.1093/emph/eoy007

**Published:** 2018-02-27

**Authors:** Amanda J Lea, Jenny Tung, Elizabeth A Archie, Susan C Alberts

**Affiliations:** 1 Department of Biology, Duke University, Durham, NC 27708, USA; 2 Institute of Primate Research, National Museums of Kenya, Karen, Nairobi, Kenya; 3 Duke University Population Research Institute, Duke University, Durham, NC 27708, USA; 4 Department of Evolutionary Anthropology, Duke University, Durham, NC 27708, USA; 5 Department of Biological Sciences, University of Notre Dame, Notre Dame, IN 46556, USA

**Keywords:** developmental plasticity, early life effects, gene-environment interaction, epigenetics, evolution of plasticity, developmental origins of health and disease

We are grateful to the commenters for their thoughtful reading of our review. Our main goals were to draw attention to the parallel significance of developmental plasticity research in evolutionary and health-related fields, and to highlight shared gains that would result from addressing outstanding questions. In doing so, we hoped to spark excitement about multi-disciplinary approaches to understanding developmental plasticity, and to emphasize that evolutionary research can inform questions of import to human health researchers and vice versa.

We focus our response on three themes that were touched on in different ways by different commenters. First, Wells, Michels and Kuzawa all discuss the *timing of early life environmental effects* and the *role of parents*, together raising several key points for discussion: (i) What is the definition of ‘early life’? (ii) When in early life is plasticity greatest? and (iii) To what degree do parents buffer or mediate the impact of ecological stressors on their offspring?

Second, the comments of Michels and Watve highlight a common misconception about the two groups of models proposed to explain developmental plasticity, namely that predictive models view developmental plasticity as an adaptation, while constraints models do not. We now clarify that *both* classes of models posit that *developmental plasticity is adaptive and has evolved through natural selection*. Below we discuss this underappreciated point; further, we address criticisms of common tests of these hypotheses brought up by Watve.

Third, Kirchengast emphasizes the role of developmental plasticity in generating variation in human life history traits. In doing so, she points out that human and non-human mammals exhibit dramatic life history differences, which calls into question the degree to which *animal models are useful for understanding developmental plasticity in humans*.

We address each of these three points below. In addition, we continue to emphasize that inter- or multi-disciplinary research approaches should be used to tackle outstanding questions about developmental plasticity. In response to Stearns’ query about research priorities, we end by enumerating three areas that are well-positioned to benefit from this perspective.

## THE TIMING OF EARLY LIFE ENVIRONMENTAL EFFECTS

In our review, we adopt the permissive definition of ‘early life’ proposed by Lindstrom [1, 2], which defines early life as the period between conception and reproductive maturation. We choose this broad definition because research from humans and other long-lived mammals has shown that experiences *in utero* [3–5], during infancy [6–9] and throughout childhood [10–14] can all shape fitness-related traits expressed in adulthood. In some cases, environmental effects on later life traits only manifest if the exposure occurs at a specific point in development (often referred to as a ‘critical period’). For example, *in utero* exposure to the Dutch Hunger Winter—a severe famine that occurred in the Netherlands during World War II—led to changes in DNA methylation, obesity and coronary heart disease risk in adulthood, but only for individuals exposed during early gestation [15–18]. However, many early life effects are not limited to critical periods. For instance, the effects of early life undernutrition on stature and work capacity in humans appear to be graded, with effect sizes that remain substantial across a range of post-natal developmental stages [13, 19]. This mix of effect types leaves open the question of when, and for what phenotypes, critical periods are likely to evolve [20].

Linked to the idea of critical periods, Wells, Michels and Kuzawa all propose that, in mammals, plasticity is greatest at conception or during gestation and dwindles thereafter. However, it is not clear how the total capacity for plasticity should be measured. Hence, the belief that plasticity is maximized in the prenatal period still demands support from empirical evidence. Michels provides a citation to [21], which includes a heuristic diagram (Fig. 3) suggesting that epigenetic plasticity is greatest during early development and decreases thereafter. This idea is consistent with the fact that both maternal and paternal genomes undergo extensive demethylation following fertilization, followed by *de novo* methylation and faithful replication of DNA methylation patterns across subsequent mitotic cell divisions. Environmental challenges that perturb the *de novo* methylation process are thus thought to affect life-long methylation patterns [22–25]. However, it is also clear that considerable plasticity is retained post-gestation [13, 19]. This is certainly true in the case of DNA methylation, where epigenetic remodeling in response to environmental stimuli in adulthood is now well-described, even in non-dividing cell types [26–30]. Therefore, we challenge researchers to test further the idea that plasticity is greatest during gestation, using empirical data. For example, a recent study [31] used data from 21 mammalian species to conclude that maternal stress during early gestation had stronger effects on offspring growth rates than maternal stress during late gestation. Similar meta-analytic approaches, as well as work in model organisms or longitudinally-followed animals, could be leveraged to understand whether maximal plasticity during gestation is common, including the types of species, exposures and fitness-related traits for which this model does or does not hold.

## THE ROLE OF PARENTS IN EARLY LIFE EFFECTS

Kuzawa and Wells both rightfully point out that our review does not address the ways in which parents mediate environmental effects on their offspring. Wells focuses on the fact that, during gestation and to some degree lactation, ‘there is no ecological stress that is not mediated by maternal phenotype’. Thus, mothers may buffer their offspring from environmental challenges, and maternal quality or condition can influence their ability to do so. In line with this idea, Kuzawa argues that maternal condition at the time of conception (which integrates maternal experiences over her life-time) may be an important early life environmental cue for the offspring. We agree that mothers are an integral mediator and component of the environment their offspring experience, and we acknowledge Kuzawa’s conclusion that maternal condition could provide ‘a reliable cue of long-term local conditions’. However, long-lived animals like humans and other large mammals will also typically experience unpredictable, year-to-year heterogeneity in ecological conditions (e.g. rainfall or food availability) [6, 7, 32, 33]. Further, such animals are often able to leave unfavorable environments and seek new ones [34]. Both of these factors will limit the predictive power of long-term maternal quality for the offspring’s future environment. Importantly, it follows that predictive plasticity will rarely evolve, in long-lived organisms, in response to maternal condition.

However, maternal quality is undoubtedly important, and we [6] and others [35] have shown that maternal characteristics can buffer offspring from the costs associated with other sources of early adversity. Understanding inter-generational effects, using physiological data, fitness-related measures or epigenomic data (as suggested by Stearns and others [36–39]), should be a priority for future research. Specifically, in systems where animals are tracked longitudinally and across generations, researchers could ask whether variation in the offspring’s epigenome is better predicted by: (i) cumulative maternal experiences prior to pregnancy (to test the ideas raised by Kuzawa); (ii) the interaction between maternal condition and ecological challenges during pregnancy (to test the hypothesis that high quality mothers ‘protect’ offspring against external stressors) or (iii) main effects of the offspring’s *in utero* or infant environment. Such work would provide much-needed insight about the timing of early life effects and the role of parents, in addition to unifying proximate and ultimate questions.

## DEVELOPMENTAL PLASTICITY EVOLVED THROUGH NATURAL SELECTION

Michels writes that developmental plasticity contradicts evolutionary principles and is not adaptive in many cases. Similarly, Watve focuses on the need to identify ‘the adaptive component of the phenotype’ that is induced by an early life exposure (implying that only certain components of developmental plasticity are selectively advantageous). In response to these comments, we emphasize that *both predictive models and constraints models* rely on adaptation via natural selection to explain early life effects. In the case of developmental constraints, fitness is expected to be higher in individuals that are plastic (i.e. can re-allocate investment to promote survival in poor environments) than in those that are not. Specifically, constraints-induced plasticity is *selectively advantageous* because individuals that exhibit the constrained phenotype outperform individuals who exhibit no plasticity. Indeed, the original formulation of the thrifty phenotype hypothesis implies exactly this type of naturally-selected re-allocation (specifically, to promote survival even if it means impaired pancreatic function [40, 41]); only later versions of this hypothesis invoked maternal forecasting as a key component of an adaptive outcome [42].

Watve also suggests that the test of predictive versus constraints models that we describe in the review (and that has been proposed by many others [6, 32, 33, 43, 44]) is insufficient. Specifically, we argue that researchers must compare the fitness of individuals born in high-quality environments with those born in low-quality environments, when each set of individuals experience both high- and low- quality conditions as adults. Under a predictive model, fitness will be maximized when individuals encounter matched early life and adult environments, whereas under a constraints model, individuals born in high-quality environments will consistently outperform individuals born in low-quality environments. We refer to this ‘fully factorial’ design as the critical test of predictive versus constraints models, because the two outcomes are mutually exclusive (put another way, one involves crossing reaction norms while the other does not, see Ref. [43]).

Recapitulating ideas from [45], Watve suggests that an additional measure is needed to provide support for predictive models: namely, data on whether a focal individual developed the phenotype that is ‘expected’ following an early life insult and whether those that developed the expected phenotype had higher fitness than those that did not. For example, in the classic case of *Daphnia*, early life exposure to predator kairomones leads to the development of defensive body structures [46, 47]. To satisfy Watve’s criterion, we would need data on whether or not an individual successfully produced the defensive phenotype following a predator encounter and whether doing so resulted in higher fitness than failing to do so. Here, Watve (and Ref [45]) would expect higher fitness in predator-rich adult environments for individuals that developed the defensive morphology relative to those that did not.

We agree with this point in principle, although performing such tests in practice is complicated by other inter-individual differences that may affect an organism’s capacity to respond (e.g. if only animals in better condition exhibit the expected plastic change). In addition, we emphasize that tests of predictive versus constraints models must be conducted not only in species like *Daphnia* with discrete polymorphisms (which are relatively rare in mammals), but also in species that produce graded, continuous responses to early environments (see Fig. 1C in our review) [6, 32, 48]. Critically, individuals of such species often vary in the extent of their response to the early environment, and no single phenotype is ‘expected.’ However, the predictions of the PAR should still hold: fitness should be maximized when individuals encounter adult environments that match their early life conditions, even if the slopes of individual reaction norms vary (for example, depending on the magnitude of each individual’s phenotypic response to early conditions). Going forward, it will be extremely important to understand inter-individual variation in the magnitude of plastic responses, including its causes (e.g. the contribution of genetic variation, which we discuss at length in our review) and evolutionary consequences.

## ARE ANIMAL SYSTEMS USEFUL FOR UNDERSTANDING DEVELOPMENTAL PLASTICITY IN HUMANS?

Several aspects of human biology are often described as unique from other animals (including in the comments of Kirchengast), raising the question of whether work in animal systems is relevant for understanding the evolution, genetic basis and molecular mechanisms underlying early life effects in humans. For example, humans have a very long, slow, late-maturing life history, with the consequence that human fitness is more heavily influenced by survival to adulthood than variation in fertility [49]. Humans have undergone selection for both a narrow pelvis (to facilitate bipedalism) and exhibit a large neonatal brain [50], leading to limits on offspring growth *in utero* (a phenomenon known as ‘maternal constraint’ [51, 52]). Finally, the environments that humans currently inhabit are dramatically different from those in which we evolved, resulting in a ‘mismatch’ between our evolved plastic responses and current environmental variation [53].

However, in all these cases, humans lie on the same trait continua as other large mammals, although they are relatively far along on each trait continuum. For instance, all long, slow life histories result in fitness outcomes that are more sensitive to survival than to other components of fitness [54–57]. This sensitivity stems from a late age at maturity in humans as well as other long-lived mammals, and not from other features of the human life history such as the post-reproductive lifespan [49, 55]. With respect to the relationship between maternal and offspring body and brain size, what sets humans apart from other primates is not our relative brain size at birth, but the fact that we exhibit more extended brain growth in the post-natal period than most other primates, indicating potentially greater nutritional constraints on post-natal development [50, 58]. In addition, limits on offspring size at birth because of constraints on the mother are seen in a range of taxa beyond primates, for example in equids (where the idea originated [59]). Finally, many natural animal populations are currently exposed to highly novel conditions as a result of human-induced environmental change [60, 61]; thus, ‘mismatch’ is an experience that is currently shared between humans and many other extant organisms.

The observation that humans lie along a shared continuum with other animals, rather than being biologically unique, is an important point that underlies essentially all research on animal models for human traits. It also suggests that the rules and processes governing the evolution of human developmental plasticity are likely to be shared with other mammalian taxa. Further, the place of humans towards the end of many (but not all) trait continua points to animals that similarly lie towards the ends of these continua as especially appropriate models for human development. Comparative cross-taxon studies of developmental plasticity can thus provide key insight into common evolutionary processes and molecular mechanisms.

## CONCLUDING REMARKS

We conclude by addressing Stearns’ call for clear priorities for empirical research. Specifically, we propose three high-priority research areas. First, repeated critical tests that clearly distinguish between predictive versus constraint models will be essential for correcting the common misconception that development constraints are ‘maladaptive’, and for improving our understanding of the evolutionary logic through which associations between early adversity and human health arise. Second, identifying loci that underlie developmental plasticity, and measuring variation in these regions of the genome both within and between species, will propel our understanding of early life effects forward. Specifically, doing so will provide insight into the evolutionary origins of developmental plasticity, as well as the degree to which plastic responses are shared versus taxon-specific. Third, understanding the epigenetic mechanisms that allow early environmental variation to produce diverse phenotypes from static gene sequences is already a research priority in human genetics and molecular biology. As these mechanisms become better understood, they will provide key empirical grounding for theoretical models of the evolution of plasticity. They will also be useful for investigating factors that explain heterogeneity in the response to the same environmental challenges (e.g. across species, individuals or cell types).

## FUNDING

This work was supported by NIH P01-AG031719 and NSF IOS-1456832 to S.C.A., R21-AG049936 and 1R01GM102562 to J.T., NSF BCS-1455808 to J.T. and A.J.L., a Leakey Foundation Research Grant to A.J.L. and S.C.A., and a Triangle Center for Evolutionary Medicine Fellowship to A.J.L.

**Conflict of interest**: None declared.

## References

[1] Lindström J. Early development and fitness in birds and mammals. Trends Ecol Evol 1999;14:343–8. 10441307 10.1016/s0169-5347(99)01639-0

[2] Henry CJK, Ulijaszek SJ, (eds.). Long-Term Consequences of Early Environment. New York, New York: Cambridge University Press, 1996.

[3] Gluckman PPD, Hanson M, Cooper C Effect of in utero and early-life conditions on adult health and disease. N Engl J Med 2008;359:61–73. 18596274 10.1056/NEJMra0708473 PMC3923653

[4] Jones P. Schizophrenia after prenatal exposure to the Dutch hunger winter of 1944-1945. Arch Gen Psychiatry 1994;51:333–4. 8161294 10.1001/archpsyc.1994.03950040077010

[5] de Rooij SR, Painter RC, Phillips DIW Impaired insulin secretion after prenatal exposure to the Dutch famine. Diabetes Care 2006;29:1897–901. 16873799 10.2337/dc06-0460

[6] Lea AJ, Altmann J, Alberts SC et al. Developmental constraints in a wild primate. Am Nat 2015;185:809–21. 25996865 10.1086/681016 PMC4541805

[7] Hayward AD, Rickard IJ, Lummaa V. Influence of early-life nutrition on mortality and reproductive success during a subsequent famine in a preindustrial population. Proc Natl Acad Sci 2013;110:13886–91. 23918366 10.1073/pnas.1301817110 PMC3752237

[8] Mumby HS, Mar KU, Hayward AD, Nature Publishing Group Elephants born in the high stress season have faster reproductive ageing. Sci Rep 2015;5:1–11. 10.1038/srep13946 PMC4568471 26365592

[9] Nussey DH, Kruuk LEB, Morris A Environmental conditions in early life influence ageing rates in a wild population of red deer. Curr Biol 2007;17:R1000–1. 18054756 10.1016/j.cub.2007.10.005

[10] Barboza Solís C, Kelly-Irving M, Fantin R Adverse childhood experiences and physiological wear-and-tear in midlife: findings from the 1958 British birth cohort. Proc Natl Acad Sci 2015;112:E738–46. 25646470 10.1073/pnas.1417325112 PMC4343178

[11] Galobardes B, Smith GD, Lynch JW. Systematic review of the influence of childhood socioeconomic circumstances on risk for cardiovascular disease in adulthood. Ann Epidemiol 2006;16:91–104. 16257232 10.1016/j.annepidem.2005.06.053

[12] Smith G, Hart C, Blane D Adverse socioeconomic conditions in childhood and cause specific adult mortality: prospective observational study. BMJ Br Med J 1998;316:1631–5. 9603744 10.1136/bmj.316.7145.1631 PMC28561

[13] Chen Y, Zhou L-A. The long-term health and economic consequences of the 1959–1961 famine in China. J Health Econ 2007;26:659–81. 17289187 10.1016/j.jhealeco.2006.12.006

[14] Wachs TD, Georgieff M, Cusick S et al. Issues in the timing of integrated early interventions: contributions from nutrition, neuroscience, and psychological research. Ann N Y Acad Sci 2014;1308:89–106. 24354763 10.1111/nyas.12314 PMC4075015

[15] Heijmans BT, Tobi EW, Stein AD Persistent epigenetic differences associated with prenatal exposure to famine in humans. Proc Natl Acad Sci 2008;105:17046–9. 18955703 10.1073/pnas.0806560105 PMC2579375

[16] Tobi EW, Lumey LH, Talens RP DNA methylation differences after exposure to prenatal famine are common and timing- and sex-specific. Hum Mol Genet 2009;18:4046–53. 19656776 10.1093/hmg/ddp353 PMC2758137

[17] Tobi EW, Goeman JJ, Monajemi R DNA methylation signatures link prenatal famine exposure to growth and metabolism. Nat Commun 2014;5:5592–13. 25424739 10.1038/ncomms6592 PMC4246417

[18] Roseboom T, de Rooij S, Painter R. The Dutch famine and its long-term consequences for adult health. Early Hum Dev 2006;82:485–91. 16876341 10.1016/j.earlhumdev.2006.07.001

[19] Hoddinott J, Alderman H, Behrman JR The economic rationale for investing in stunting reduction. Matern Child Nutr 2013;9:69–82. 24074319 10.1111/mcn.12080 PMC6860695

[20] Wells JCK. Adaptive variability in the duration of critical windows of plasticity. Evol Med Public Heal 2014;2014:109–21. 10.1093/emph/eou019 PMC4148720 25095791

[21] Godfrey KM, Lillycrop KA, Burdge GC Epigenetic mechanisms and the mismatch concept of the developmental origins of health and disease. Pediatr Res 2007;61:5R–36. 10.1203/pdr.0b013e318045bedb 17413851

[22] Suzuki MM, Bird A. DNA methylation landscapes: provocative insights from epigenomics. Nat Rev Genet 2008;9:465–76. 18463664 10.1038/nrg2341

[23] Klose RJ, Bird AP. Genomic DNA methylation: the mark and its mediators. Trends Biochem Sci 2006;31:89–97. 16403636 10.1016/j.tibs.2005.12.008

[24] Jaenisch R, Bird A. Epigenetic regulation of gene expression: how the genome integrates intrinsic and environmental signals. Nat Genet 2003;33:245–54. 12610534 10.1038/ng1089

[25] Jirtle RL, Skinner MK. Environmental epigenomics and disease susceptibility. Nat Rev Genet 2007;8:253–62. 17363974 10.1038/nrg2045 PMC5940010

[26] Pacis A, Tailleux L, Lambourne J Bacterial infection remodels the DNA methylation landscape of human dendritic cells. Genome Res 2015;25:1801–11. 26392366 10.1101/gr.192005.115 PMC4665002

[27] Barrès R, Yan J, Egan B Acute exercise remodels promoter methylation in human skeletal muscle. Cell Metab 2012;15:405–11. 22405075 10.1016/j.cmet.2012.01.001

[28] Tung J, Barreiro LB, Johnson ZP Social environment is associated with gene regulatory variation in the rhesus macaque immune system. Proc Natl Acad Sci 2012;109:6490–5. 22493251 10.1073/pnas.1202734109 PMC3340061

[29] Netea MG, Joosten LAB, Latz E Trained immunity: a program of innate immune memory in health and disease. Science 2016;352:aaf1098. 27102489 10.1126/science.aaf1098 PMC5087274

[30] Miller CA, Sweatt JD. Covalent modification of DNA regulates memory formation. Neuron 2007;53:857–69. 17359920 10.1016/j.neuron.2007.02.022

[31] Berghänel A, Heistermann M, Schülke O et al. Prenatal stress accelerates offspring growth to compensate for reduced maternal investment across mammals. Proc Natl Acad Sci 2017;114:E10658–66. 29180423 10.1073/pnas.1707152114 PMC5740685

[32] Douhard M, Plard F, Gaillard J-M Fitness consequences of environmental conditions at different life stages in a long-lived vertebrate. Proc R Soc London, Ser B 2014;281:20140276. 10.1098/rspb.2014.0276 PMC4024291 24789898

[33] Hayward AD, Lummaa V. Testing the evolutionary basis of the predictive adaptive response hypothesis in a preindustrial human population. Evol Med Public Heal 2013;2013:106–17. 10.1093/emph/eot007 PMC3868390 24481192

[34] Eriksson A, Betti L, Friend AD Late Pleistocene climate change and the global expansion of anatomically modern humans. Proc Natl Acad Sci 2012;109:16089–94. 22988099 10.1073/pnas.1209494109 PMC3479575

[35] Chen E, Miller GE, Kobor MS , Nature Publishing Group. Maternal warmth buffers the effects of low early-life socioeconomic status on pro-inflammatory signaling in adulthood. Mol Psychiatry 2011;16:729–37. 20479762 10.1038/mp.2010.53 PMC2925055

[36] Pesonen A-K, Räikkönen K. The lifespan consequences of early life stress. Physiol Behav Elsevier B.V 2012;106:722–7. 10.1016/j.physbeh.2011.10.030 22079583

[37] Gertz J, Varley KE, Reddy TE Analysis of DNA methylation in a three-generation family reveals widespread genetic influence on epigenetic regulation. PLoS Genet 2011;7:e1002228. 21852959 10.1371/journal.pgen.1002228 PMC3154961

[38] Godfrey KM, Gluckman PD, Hanson MA. Developmental origins of metabolic disease: life course and intergenerational perspectives. Trends Endocrinol Metab 2010;21:199–205. 20080045 10.1016/j.tem.2009.12.008

[39] Heard E, Martienssen RA. Transgenerational Epigenetic Inheritance: myths and mechanisms. Cell 2014;157:95–109. 24679529 10.1016/j.cell.2014.02.045 PMC4020004

[40] Neel J. Diabetes mellitus: a ‘Thrifty’ genotype rendered detrimental by ‘Progress’? Am J Hum Genet 1962;14:353–62. 13937884 PMC1932342

[41] Hales C, Barker D. Type 2 (non-insulin-dependent) diabetes mellitus: the thrifty phenotype hypothesis. Diabetologia 1992;35:595–601. 1644236 10.1007/BF00400248

[42] Hales C, Barker D. The thrifty phenotype hypothesis. Br Med Bull 2001;60:5–20. 11809615 10.1093/bmb/60.1.5

[43] Monaghan P. Early growth conditions, phenotypic development and environmental change. Philos Trans R Soc Lond B Biol Sci 2008;363:1635–45. 18048301 10.1098/rstb.2007.0011 PMC2606729

[44] Uller T, Nakagawa S, English S. Weak evidence for anticipatory parental effects in plants and animals. J Evol Biol 2013;26:2161–70. 23937440 10.1111/jeb.12212

[45] Nettle D, Bateson M. Adaptive developmental plasticity: what is it, how can we recognize it and when can it evolve? Proc R Soc B 2015;282:20151005. 10.1098/rspb.2015.1005 PMC4528519 26203000

[46] Laforsch C, Beccara L, Tollrian R. Inducible defenses: the relevance of chemical alarm cues in *Daphnia*. Limnol Oceanogr 2006;51:1466–72.

[47] Boersma M, Spaak P, De Meester L. Predator-mediated plasticity in morphology, life history, and behavior of Daphnia: the uncoupling of responses. Am Nat 1998;152:237–48. 18811388 10.1086/286164

[48] Pigeon G, Festa-Bianchet M, Pelletier F. Long-term fitness consequences of early environment in a long-lived ungulate. Proc R Soc B 2017;284:20170222. 10.1098/rspb.2017.0222 PMC5413925 28424347

[49] Jones JH. The force of selection on the human life cycle. Evol Hum Behav 2009;30:305–14. 22003281 10.1016/j.evolhumbehav.2009.01.005 PMC3193054

[50] Martin R. The evolution of human reproduction: a primatological perspective. Yearb Phys Anthropol 2007;134:59–84. 10.1002/ajpa.20734 18046752

[51] Kuzawa CW, Eisenberg DTA. Intergenerational predictors of birth weight in the Philippines: correlations with mother’s and father’s birth weight and test of maternal constraint. PLoS One 2012;7:e40905–9. 22848409 10.1371/journal.pone.0040905 PMC3407139

[52] Rickard IJ, Courtiol A, Prentice AM Intergenerational effects of maternal birth season on offspring size in rural Gambia. Proc R Soc B Biol Sci 2012;279:4253–62. 10.1098/rspb.2012.1363 PMC3441076 22896641

[53] Gluckman PD, Hanson MT. Mismatch: The Lifestyle Diseases Timebomb. Oxford: Oxford University Press, 2008.

[54] Jones OR, Gaillard JM, Tuljapurkar S Senescence rates are determined by ranking on the fast-slow life-history continuum. Ecol Lett 2008;11:664–73. 18445028 10.1111/j.1461-0248.2008.01187.x

[55] Jones JH. Primates and the evolution of long-slow life histories. Curr Biol 2011;21:R708–17. 21959161 10.1016/j.cub.2011.08.025 PMC3192902

[56] Mcdonald DB, Station AB, Placid L. Demographic consequences of sexual selection in the long-tailed manakin. Behav Ecol 1993;4:297–309.

[57] Alberts SC, Altmann J. Intraspecific variability in fertility and offspring survival in a nonhuman primate: behavioral control of ecological and social sources In: Wachter KW, Bulatao RA (eds.). Offspring: The Biodemography of Fertility and Family Behavior. Washington, DC: National Academy Press, 2003, 140–69.

[58] Martin RD. Scaling of the mammalian brain: the maternal energy hypothesis. News Physiol Sci 1996;11:149–56.

[59] Walton A, Hammond J. The maternal effects on growth and conformation in Shire Horse-Shetland Pony crosses. Proc R Soc Ser B 1938;125:311.

[60] Sih A, Ferrari MCO, Harris DJ. Evolution and behavioural responses to human-induced rapid environmental change. Evol Appl 2011;4:367–87. 25567979 10.1111/j.1752-4571.2010.00166.x PMC3352552

[61] Sih A, Bolnick DI, Luttbeg B Predator-prey naïveté, antipredator behavior, and the ecology of predator invasions. Oikos 2010;119:610–21

